# TLR4/NF-***κ***B-Responsive MicroRNAs and Their Potential Target Genes: A Mouse Model of Skeletal Muscle Ischemia-Reperfusion Injury

**DOI:** 10.1155/2015/410721

**Published:** 2015-01-26

**Authors:** Johnson Chia-Shen Yang, Shao-Chun Wu, Cheng-Shyuan Rau, Yi-Chun Chen, Tsu-Hsiang Lu, Yi-Chan Wu, Siou-Ling Tzeng, Chia-Jung Wu, Ching-Hua Hsieh

**Affiliations:** ^1^Department of Plastic and Reconstructive Surgery, Kaohsiung Chang Gung Memorial Hospital and Chang Gung University College of Medicine, No. 123, Ta-Pei Road, Niao-Song District, Kaohsiung City 833, Taiwan; ^2^Department of Anesthesiology, Kaohsiung Chang Gung Memorial Hospital and Chang Gung University College of Medicine, No. 123 Ta-Pei Road, Niao-Song District, Kaohsiung City 833, Taiwan; ^3^Department of Neurosurgery, Kaohsiung Chang Gung Memorial Hospital and Chang Gung University College of Medicine, No. 123 Ta-Pei Road, Niao-Song District, Kaohsiung City 833, Taiwan

## Abstract

*Background*. The aim of this study was to profile TLR4/NF-*κ*B-responsive microRNAs (miRNAs) and their potential target genes in the skeletal muscles of mice following ischemia-reperfusion injury. *Methods*. Thigh skeletal muscles of C57BL/6, *Tlr4* ^−/−^, and *NF-*κ*B* ^−/−^ mice isolated based on femoral artery perfusion were subjected to ischemia for 2 h and reperfusion for 0 h, 4 h, 1 d, and 7 d. The muscle specimens were analyzed with miRNA arrays. Immunoprecipitation with an argonaute 2- (Ago2-) specific monoclonal antibody followed by whole genome microarray was performed to identify mRNA associated with the RNA-silencing machinery. The potential targets of each upregulated miRNA were identified by combined analysis involving the bioinformatics algorithm miRanda and whole genome expression. *Results*. Three TLR4/NF-*κ*B-responsive miRNAs (miR-15a, miR-744, and miR-1196) were significantly upregulated in the muscles following ischemia-reperfusion injury. The combined in silico and whole genome microarray approaches identified 5, 4, and 20 potential target genes for miR-15a, miR-744, and miR-1196, respectively. Among the 3 genes (*Zbed4, Lrsam1,* and *Ddx21*) regulated by at least 2 of the 3 upregulated miRNAs, *Lrsam1* and *Ddx21* are known to be associated with the innate immunity pathway. *Conclusions*. This study profiled TLR4/NF-*κ*B-responsive miRNAs and their potential target genes in mouse skeletal muscle subjected to ischemia-reperfusion injury.

## 1. Introduction

Despite extensive experimental work directed toward the treatment and prevention of established skeletal muscle ischemia-reperfusion injury (IRI), the clinical outcome has not appreciably changed over the past few decades [1]. Better understanding of the pathophysiological processes occurring within skeletal muscle in IRI may enable identification of potential therapeutic targets. Recent studies have indicated the involvement of toll-like receptors (TLRs) in the pathogenesis of IRI in various organs and systems [2]. Recent observations have indicated that some TLRs also alert the host to the presence of tissue damage and become activated by endogenous molecules released from damaged ischemic tissues [3, 4]. Stimulation of TLRs upon ligand recognition triggers downstream signaling cascades, which culminates in the activation of nuclear factor-*κ*B (NF-*κ*B) and activator protein 1 (AP-1) and results in the release of various proinflammatory cytokines such as IL-6, IL-1, and TNF-*α*. NF-*κ*B is thought to play an important role in the activation of genes expressed in response to IRI, and regulation of the initial phase of NF-*κ*B activation affords physiological protection against severe ischemic stress [5, 6].

TLRs 1–9 have been found to be expressed in skeletal muscles [7, 8]. In patients with critical limb ischemia, expression of the TLR 2, 4, and 6 proteins was observed in gastrocnemius muscle biopsy samples [9]. TLR4, a TLR family member, is considered a central mediator of IRI-related inflammation and organ injury, along with a family of putative danger signal molecules, which includes hyaluronic acid, heparan sulfate, fibrinogen, HMGB1, and heat shock proteins [10–12]. TLR4 also participates in the recognition of endogenous proteins released from damaged tissues in hemorrhagic shock [13] and in cardiac [14], renal [15], and hepatic [16] IRI models. Various studies have shown that TLR4-deficient mice have lesser myocardial infarct size than wild-type control animals [14, 17]. Other studies have suggested that smaller myocardial infarctions in TLR4-deficient mice can be attributed to reduced neutrophil infiltration and NF-*κ*B activation followed by decrease in the levels of the inflammatory cytokines IL-1*β* and IL-6 as well as of monocyte chemotactic factor-1 [14, 17]. TLR4/NF-*κ*B signaling has been suggested to play a pivotal role in mediating hindlimb IRI through decrease in neutrophil extracellular traps, which may contribute to muscle fiber injury [18]. In addition, blockade with TLR4 antagonists has been shown to have a beneficial effect in myocardial infarction [19, 20].

MicroRNAs (miRNAs) are small noncoding RNAs belonging to a novel class of negative regulators that control gene expression by imperfect base pairing with the 3′ untranslated region (3′UTR) of a target mRNA, leading to inhibition of translation or mRNA degradation [21]. Both basic and clinical studies have suggested that miRNAs are important regulators of cell differentiation, growth, proliferation, and apoptosis [22, 23]. These observations are probably not surprising as bioinformatics predictions indicate that mammalian miRNAs can regulate approximately 60% of all protein-coding genes [24, 25]. miRNAs also play important roles in the regulation of the innate immune system [26]. The end result of the TLR signaling pathways is the activation of proinflammatory transcription factors that enhance transcription of RNA polymerase II-sensitive genes encoding cytokines, chemokines, and antimicrobial factors. Because miRNAs are also transcribed by RNA polymerase II [27, 28], it stands to reason that miRNAs themselves are targets of TLR signaling pathways, especially considering that the expression of most of the TLR-responsive miRNAs described so far depends on NF-*κ*B activity [29]. In addition, miRNAs not only provide a link between the innate and adaptive immune signaling pathways but also play a role in controlling the switch from strong early proinflammatory responses to the resolution phase of the inflammatory process [29]. In addition, dysregulated miRNA expression has been implicated in mediating IRI in the heart, liver, and kidney [30–34]. Because TLRs are activated in sterile inflammation during IRI, regulation of TLR signaling provides an opportunity to control the IRI pathophysiological process and reduce cell damage. This study was designed to investigate the involvement of the TLR4/NF-*κ*B pathway in miRNA expression during IRI. We used miRNA array analysis to identify the miRNA-regulated target genes by using a combined approach involving prediction algorithms and a whole genome microarray experiment coupled with an argonaute 2 (Ago2) ribonucleoprotein immunoprecipitation- (RIP-) chip.

## 2. Materials and Methods

### 2.1. Animal Experiments and Tissue Preparation

**Figure 1:**
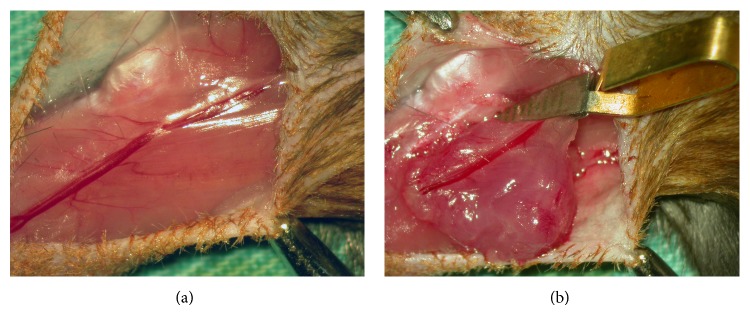
Model of ischemia-reperfusion injury on the isolated skeletal muscle. (a) Mouse quadriceps muscle was perfused based on the overlying femoral artery. (b) After isolation of the muscle, ischemia was induced by placing a microvascular clamp across the proximal vascular pedicle for 2 h.

Male C57BL/6 mice (age, 10–12 weeks; weight, 22–35 g) were purchased from BioLasco (Taiwan). *Tlr*4^−/−^ (C57BL/10ScNJ) and *NF*-*κB* ^−/−^ (B6.Cg-Nfkb1tm1Bal/J) mice were purchased from Jackson Laboratory (Bar Harbor, ME, USA). All the housing conditions and the surgical procedures, analgesia, and assessments were in accordance with national and institutional guidelines and an AAALAC-accredited SPF facility was used. The animal protocols were approved by the IACUC of Chang Gung Memorial Hospital. Mice were anesthetized by intraperitoneal injection of an anesthetic cocktail consisting of 0.1 mg/g ketamine and 0.01 mg/g xylazine (0.01 mL/g body weight). The anesthetized mice were restrained in a supine position on a heated pad to maintain the body temperature at 37°C. The quadriceps muscle was perfused based on the femoral artery (Figure 1(a)) and carefully separated away from the femoral bone and the underlying adductor muscle group. In the ischemic group, ischemia was induced by placing a microvascular clamp carefully across the proximal site of the vascular pedicle (Figure 1(b)) for 2 h after which the clamp was removed. Good vascular flow through the pedicle was verified under direct magnified vision. In the sham-operated control group, the muscle was isolated without inducing ischemia with a microvascular clamp. The incision wound was closed with interrupted sutures (4-0 nylon sutures), and the animals were allowed to awaken during the remaining reperfusion time. The harvested muscles were frozen in isopentane chilled in liquid nitrogen and stored at −80°C. For the miRNA array experiments, isolated skeletal muscles of C57BL/6 mice after 2 h of ischemia and 0 h, 4 h, 1 d, and 7 d of reperfusion and of *Tlr*4^−/−^ and *NF*-*κB* ^−/−^ mice after 2 h of ischemia and 1 d of reperfusion were used in 3 replicate experiments. For the whole genome array experiments, isolated skeletal muscles of C57BL/6 mice after 2 h of ischemia and 24 h of reperfusion were used in 2 replicate experiments.

### 2.2. RNA Isolation

Total RNA was extracted using the mirVana miRNA Isolation kit (Ambion, Austin, TX, USA). For the miRNA and whole genome expression analyses, the purified RNA yield was determined by absorbance at 260 nm by using an SSP-3000 Nanodrop spectrophotometer (Infinigen Biotech, City of Industry, CA, USA), and RNA quality was evaluated with a Bioanalyzer 2100 system (Agilent Technologies, Palo Alto, CA, USA).

### 2.3. miRNA Array Analysis

The Mouse & Rat miRNA OneArray v3 (Phalanx Biotech Group, Hsinchu, Taiwan) contains a total of 4104 probes, including 144 experimental control probes, 1111 unique mouse miRNA probes, and 680 rat miRNA probes, based on miRBase version 17. Mouse genome-wide miRNA microarray analysis was performed by Phalanx Biotech. Briefly, fluorescent targets were prepared from 2.5 *μ*g total RNA by using the miRNA ULS Labeling Kit (Kreatech Diagnostics, Amsterdam, The Netherlands). Labeled miRNA targets enriched using NanoSep 100 K (Pall Corporation, Port Washington, NY, USA) were hybridized to the Mouse & Rat miRNA OneArray v3 in Phalanx hybridization buffer by using the OneArray Hybridization Chamber. After overnight hybridization at 37°C, nonspecifically bound targets were removed by 3 washing steps (wash I, at 37°C for 5 min; wash II, at 37°C for 5 min and at 25°C for 5 min; and wash III, rinse 20 times at 37°C). The slides were dried by centrifugation and scanned using an Axon 4000B scanner (Molecular Devices, Sunnyvale, CA, USA). The signal intensities of Cy5 fluorescence in each spot were analyzed using the GenePix 4.1 software (Molecular Devices, CA, USA) and processed using the R program. We filtered out spots for which the flag was <0, and spots that passed these criteria were normalized using a 75% media scaling normalization method. Normalized spot intensities were converted into gene expression log_2_ ratios for the control and treatment groups. Spots with log_2_ ratios ≤−1 or ≥1 and *P* values <0.05 were selected for further analysis. The differentially expressed miRNAs were subjected to hierarchical cluster analysis by using average linkage and Pearson correlation as a measure of similarity. Five miRNAs detected by array analysis were selected and were quantified by qPCR using the Applied Biosystems 7500 Real-Time PCR System (Life Technologies) to confirm the upregulation of miRNA expression in the muscle of C57BL/6 mice after 2 h of ischemia and 1 d of reperfusion. Each miRNA expression was represented relative to the expression of small RNA 4.5S used as an internal control. The expression fold of induction was given in terms of the relative expression values obtained from 4 samples against that from the muscles of the sham control group.

### 2.4. Ribonucleoprotein Immunoprecipitation

RIP-chip, that is, immunoprecipitation of RNA-induced silencing complexes (RISC) with an Ago2-specific monoclonal antibody followed by RNA extraction and subsequent quantification of mRNAs on microarrays, has recently been utilized to identify mRNAs that are associated with the RNA-silencing machinery and are therefore targets of cellular miRNAs [35–38]. In brief, 200 *μ*g of total muscle protein was diluted with 200 *μ*L of PBS buffer (pH 7.4). For each sample, 25 *μ*L of protein A/G plus agarose (Santa Cruz) was washed with PBS and incubated with 2 *μ*g of rabbit anti-Ago2 (Abcam, MA, USA) or rabbit normal IgG (Santa Cruz) antibodies for 2 h at 4°C. The beads containing the immobilized anti-Ago2 antibody were then added to 400 *μ*L of diluted serum and incubated for 4 h at 4°C. The beads were washed 3 times with 1% NT-2 buffer (1% Nonidet P-40, 50 mM Tris-HCl, pH 7.4, 150 mM NaCl, and 2 mM EDTA) and the mixture was split in half. One-half of each sample was eluted in 2 × SDS sample buffer and subjected to SDS/PAGE and immunoblotting with a mouse anti-Ago2 antibody (Santa Cruz, CA, USA) to detect Ago2. The other half of each sample was eluted in 600 *μ*L of lysis/binding buffer from the mirVana miRNA Isolation Kit (Life Technologies, NY, USA) and processed for RNA isolation. The RNA pellet was used for oligo-dT purification and library generation.

### 2.5. Whole Genome Microarray Analysis

The microarray experiments were carried out following the manufacturer's protocols. In brief, 0.5 *μ*g of the total RNA was amplified using the Fluorescent Linear Amplification Kit (Agilent Technologies, USA) and labeled with Cy3-CTP or Cy5-CTP (CyDye, PerkinElmer, USA) during in vitro transcription. RNA from ischemic muscles was labeled with Cy5 and control RNA was labeled with Cy3. Then, 0.825 *μ*g of Cy-labeled cRNA was cut into fragments of approximately 50–100 nucleotides by incubation in the fragmentation buffer (Agilent Technologies) at 60°C for 30 min. The fragmented labeled cRNA was then pooled and hybridized to the Agilent Mouse G3 Whole Genome Oligo 8 × 60 K Microarray (Agilent Technologies) at 60°C for 17 h. After washing and drying by nitrogen gun blowing, the microarrays were scanned with the Agilent microarray scanner (Agilent Technologies) at 535 nm for Cy3 and 625 nm for Cy5. The scanned images were analyzed by Feature Extraction software 9.5.3 (Agilent Technologies); imaging analysis and a normalization software used to quantify the signal and background intensities for each feature substantially normalized the data by the rank-consistency-filtering LOWESS method.

### 2.6. Prediction of Potential miRNA Target Genes

Potential targets of the TLR4/NF-*κ*B-responsive miRNAs were identified by a combined approach based on the commonly used web tool for bioinformatics algorithms miRanda (http://www.microrna.org/microrna/home.do) and whole genome microarray hybridization analysis of dysregulated mRNAs before and after Ago2 immunoprecipitation of the muscle lysates. The in silico predicted target genes were compared to the list of 2-fold upregulated mRNA transcripts isolated by immunoprecipitation with Ago2, which implies the presence of these mRNAs in the Ago2 complex, and with the list of 2-fold downregulated mRNA transcripts identified in the whole genome microarray experiments. The genes identified by both methods were considered as potential target genes regulated by a given miRNA.

## 3. Results

### 3.1. miRNA Expression Profile

**Figure 2:**
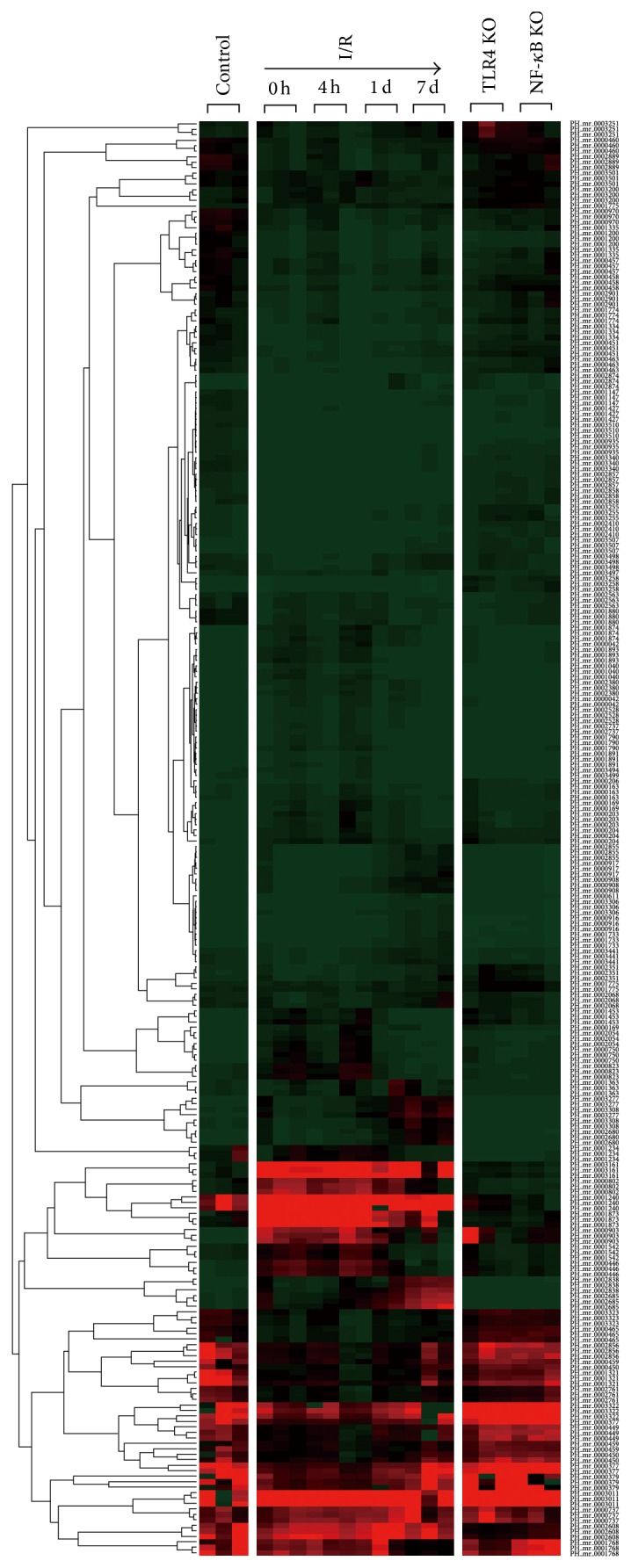
Hierarchical clustering of the expression of miRNAs. Unsupervised hierarchical clustering of miRNA differentially expressed in the IRI muscles of C57BL/6 mice (reperfusion times indicated as 0 h, 4 h, 1 d, and 7 d) and in *Tlr*4^−/−^ and *NF*-*κB* ^−/−^ mice (reperfusion 1 d) compared to sham controls (*n* = 3 for each subgroups).

miRNA expression in ischemic muscles was considered differentially regulated if the mean values for all samples demonstrated more than 2-fold difference compared with those for the control muscles, with *P* value <0.05 by miRNA array analysis. Unsupervised hierarchy clustering was used to group the experimental muscle samples of C57BL/6 mice and *Tlr*4^−/−^ and *NF*-*κB* ^−/−^ mice into separate clusters (Figure 2). The significantly upregulated miRNA targets from the muscles of C57BL/6 mice subjected to ischemia for 2 h followed by 0 h, 4 h, 1 d, or 7 d reperfusion were identified by miRNA arrays and are shown in Table 1 (detailed information in the Supplementary File 1 in Supplementary Material available online at http://dx.doi.org/10.1155/2014/410721). There were 1, 1, 128, and 59 upregulated miRNA targets in the ischemic muscles of C57BL/6 mice subjected to 0 h, 4 h, 1 d, or 7 d of reperfusion, respectively. Ischemia for 2 h without reperfusion induced upregulation of miR-21 expression that could still be detected after reperfusion for 1 and 7 d. In addition, miR-493 expression could be observed after ischemia and reperfusion for 4 h and 1 d but did not last for 7 d. When miRNA expression was compared in the muscle samples of C57BL/6 and *Tlr*4^−/−^/*NF*-*κB* ^−/−^ mice subjected to 2 h of ischemia and 1 d of reperfusion, only 3 miRNAs (miR-15a, miR-744, and miR-1196) showed significantly increased expression in C57BL/6 mice and decreased expression in *Tlr*4^−/−^/*NF*-*κB* ^−/−^ mice (Table 2). miRNA array analysis showed that the expression of miR-1196, but not miR-15a or miR-744, persisted till 7 d of reperfusion (Table 1). Microarray and qPCR results of five miRNA targets including miR-15a, miR-744, and miR-1196 in the experimental muscle of C57BL/6 mice after 2 h of ischemia and 1 d of reperfusion were in general agreement, with a Pearson correlation value of 0.912 (Supplementary File 2).

### 3.2. RIP-Chip and Whole Genome Microarray Analyses

**Figure 3:**
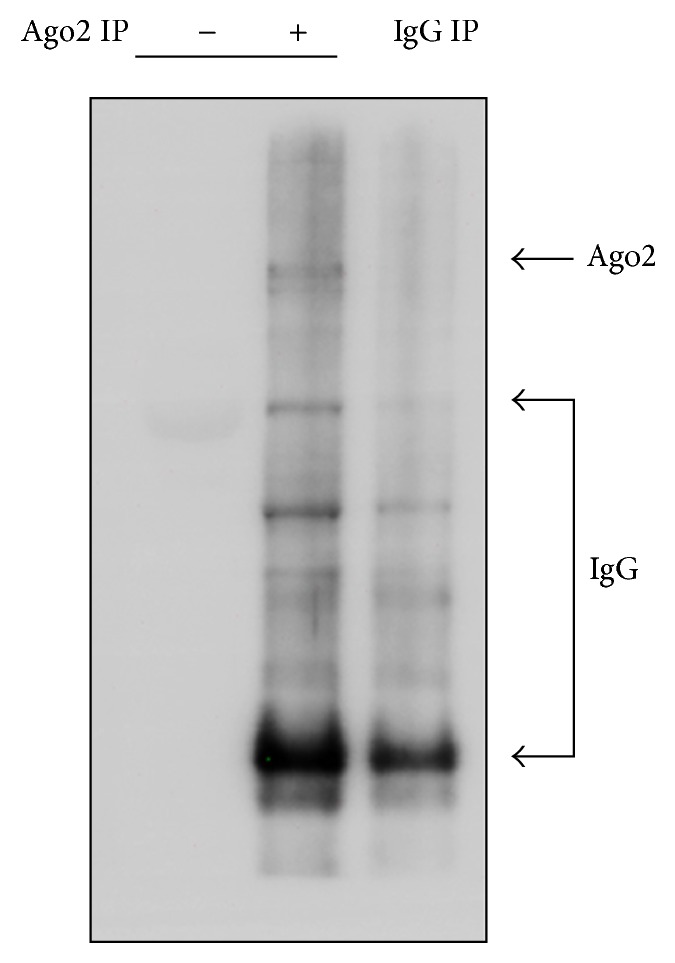
Immunoblotting analysis of muscle lysates before and after Ago2 immunoprecipitation. Total lysates (−) and precipitates with the Ago2 antibody (+) and control IgG (IgG IP) were separated by SDS-PAGE and probed with the Ago2 antibody to determine the presence of Ago2 proteins.

RIP-chip is a high-throughput method to identify mRNAs that are targeted by RNA-binding proteins (RBP) or ribonucleoproteins (RNP), such as RNA-induced silencing complex (RISC), based on immunoprecipitation (IP) of the RBP, or RNP with associated mRNAs followed by microarray [37, 39]. The protein of interest is immunoprecipitated, and the identity and relative amount of mRNA associated with it are measured on microarrays. Since miRNA function is mediated by argonaute 2 (Ago2) proteins in the RISC, an anti-Ago2 antibody was used to isolate global miRNA targets from the muscle sample under different experimental conditions, which we identified using a genome-wide comparative hybridization microarray. Immunoblotting with antibodies against Ago2 showed the presence of Ago2 proteins in immunocomplexes following immunoprecipitation (Figure 3); without immunoprecipitation, the Ago2 levels in the muscle specimens were below the limit of detection of immunoblotting. In addition, the absence of Ago2 in the negative-control IgG immunoprecipitates demonstrated the specificity of Ago2 precipitation with the anti-Ago2 antibody. Expression profiling of 2 replicates of array data of the IRI muscle samples against those of sham control mice was performed using the whole genome microarray, which showed 881 significantly (2-fold or greater) downregulated gene transcripts in the muscles of mice after ischemia and reperfusion for 1 d. In addition, the Ago2 RIP-chip assay showed 1433 significantly upregulated gene transcripts in the Ago2-pull down mRNA pool in the IRI muscles compared to those from the sham control mice.

### 3.3. Identification of Potential miRNA-Regulated Genes

Potential target genes regulated by a given miRNA were identified by in silico prediction and microarray hybridization of Ago2 coimmunoprecipitates. The combined approach showed 5, 4, and 20 potential target genes for miR-15a, miR-744, and miR-1196, respectively, in the IRI muscle samples (Table 3). Three genes were regulated by at least 2 of these 3 upregulated miRNAs; that is, zinc finger BED domain containing 4 (Zbed4) was regulated by miR-15a, miR-744, and miRR-1196; leucine-rich repeat and sterile alpha motif containing 1 (Lrsam1), by miR-15a and miR-744; and the DEAD (Asp-Glu-Ala-Asp) box polypeptide 21 (Ddx21), by miR-744 and miR-1196. The array data have been deposited in Gene Expression Omnibus (accession number GEO: GSE47730).

## 4. Discussion

In the present study, we investigated the miRNA expression profile of miRNAs in the isolated thigh skeletal muscle of mice subjected to IRI. The considerable change in miRNA expression at 1 and 7 d after IRI suggests that miRNAs may play critical roles in regulating the expression of genes in injured muscles. Among the dysregulated miRNAs, 3 TLR4/NF-*κ*B-responsive miRNAs, miR-15a, miR-744, and miR-1196, were significantly upregulated in the skeletal muscles of C57BL/6 mice following IRI, but their expression notably decreased in similarly treated *Tlr*4^−/−^/*NF*-*κB* ^−/−^ mice. In this study, there were 1, 1, 128, and 59 upregulated miRNA targets in the ischemic muscles of C57BL/6 mice subjected to 0 h, 4 h, 1 d, or 7 d of reperfusion, respectively. In ischemia, the vessel pedicle was occluded by the microclamp and cellular infiltration through the vessel pedicle to the muscle is not possible; therefore, in comparison to the very early stage of reperfusion at 0 h and 4 h, the abundant expression of miRNAs at 1 d and 7 d may be attributed to the infiltrated cells into the muscle after release of the microclamp. It had been reported that the inflammatory signals and neutrophil and monocyte infiltration were less in the TLR4 knockout mice [10] and therefore these three different expressed miRNAs between C57BL/6 as well as *Tlr*4^−/−^/*NF*-*κB* ^−/−^ mice may be also attributed to the infiltrated cells in the muscle. However, identifying the exact secreted origin of these three miRNAs require further extensive investigation. In addition, because there are signal pathways shared differently between TLR4 and NF-*κ*B, further use of MyD88 and TRIF knockout mice may help to clarify more details in the miRNAs expression through this pathway. Among these 3 upregulated miRNAs, miR-15a showed increased expression in response to myocardial IRI [40], but association of miR-744 and miR-1196 with IRI has not been previously reported. Notably, miR-744-directed posttranscriptional regulation of TGF-*β*1 is of central importance in wound healing, inflammation, and progressive tissue fibrosis, in human proximal tubular epithelial cells HK-2 [41]. miR-15a inhibits angiogenesis through direct inhibition of endogenous endothelial FGF2 and VEGF during hindlimb ischemia [42]. In human, vascular endothelial growth factor-A and AKT-3 were validated as direct targets of miR-15a, and their protein levels were reduced in miR-15a-overexpressing circulating proangiogenic cells of healthy patients and those with critical limb ischemia [43]. Overexpression of miR-15a impaired survival and migration of healthy circulating proangiogenic cells; conversely, miR-15a inhibition improved the impaired migration of circulating proangiogenic cells in critical limb ischemia [43]. Transplantation of healthy circulating proangiogenic cells engineered to overexpress anti-miR-15a improves postischemic recovery in blood flow and muscular arteriole density in mice [43]. In addition, in a mouse model of skeletal ischemia by surgical excision of the left femoral artery, histological analysis revealed a 35% increase in the capillary density of ischemic muscles compared with contralateral ones, indicative of spontaneous angiogenesis [44]. However, the exact role of miR-15a in angiogenesis after muscle IRI requires further experiments with miR-15a overexpression or inhibition for validation.

In this study, we observed that muscle ischemia for 2 h without reperfusion could induce upregulation of miR-21 expression, which could still be detected after 1 and 7 d of reperfusion. Overexpression of miR-21 has been reported to have an antiapoptotic effect and to protect against hydrogen peroxide-induced injury of cardiac myocytes via the AP-1 pathway by targeting the programmed cell death 4 (*PDCD4*) gene [45]. In addition, significant induction of miR-21 has been detected in the heart following whole-body heat shock [46]. Injection of exogenous synthetic miR-21 significantly reduces infarct size in the heart, which can be reversed by a miR-21 inhibitor [46]. In our previous study on ischemic injury in the rat gracilis muscle, we observed increased expression of miR-21 during IRI and identified 4 miR-21 potential target genes (*Nqo1, Pdpn, CXCL3, and Rad23b*) by using different prediction algorithms and monitoring miRNA and mRNA expression at different time points on a genome-wide basis [47]. Although the expression of miR-21 was not induced in *Tlr*4^−/−^/*NF*-*κB* ^−/−^ mice, miR-21 must play an important role in modulating gene expression following IRI. Furthermore, it should be noted that although there is a body of evidence for the central role of TLR4 in signaling tissue injury and this study demonstrates that the *Tlr*4^−/−^/*NF*-*κB* ^−/−^ pathway transduces signals generated by IRI, other TLR family members may participate in the recognition of endogenous molecules triggered by tissue injury [48].

In this study, 3 genes (*Zbed4*,* LRSAM1*, and* Ddx21*) were found to be regulated by at least 2 of the 3 miRNAs induced after muscle IRI. Of the 3 genes,* Lrsam1* and* Ddx21* have been reported to be associated with the innate immunity pathway. However, there was no report of these three genes related to IRI that could be found from the literature. During innate immune sensing, detection of pathogen-associated molecular patterns by TLRs typically involves leucine-rich repeats (LRRs) [49]. The LRR protein LRSAM1 (leucine-rich repeat and sterile *α*-motif containing 1) plays an essential role in antibacterial autophagy [49]. The functional siRNA approach has been used to show that knockdown of Lrsam1 results in reduction in anti-*Salmonella* autophagy [49]. Lrsam1 has the E3 ubiquitin-protein ligase domains which mediate monoubiquitination of tumor susceptibility gene (TSG) 101 at multiple sites and regulates receptor endocytosis by inactivating the ability of TSG101 to sort endocytic and exocytic cargos [50].

Ddx21 is one of the DEx(D/H) box RNA helicases that unwind RNA structure or disrupt RNA-protein interactions during cellular processes requiring modulation of RNA structures [51]. RNA helicase Ddx21 is necessary for the processing of 20S pre-rRNA into 18S rRNA and for the stability of 28S rRNA in* Xenopus* [52]. Human Ddx21 has also been shown to be critical for the production of 28S and 18S rRNAs [53]. In innate immunity, the direct interaction between the AP-1 transcription factor c-Jun and Ddx21 regulates the nucleolar localization of Ddx21 [54]. Ddx21 helicases form a complex with the adaptor molecule TRIF to sense dsRNA and activate type I interferon responses in the cytosol of dendritic cells [55]. In general, miRNAs downregulate TLR signaling by targeting downstream signaling molecules rather than shutting down the TLR pathway completely by blocking receptor expression [26]. For example, IRAK1 and TRAF6, 2 central adaptor kinases in the TLR downstream signaling cascade, are targeted by miR-146 [56]. MyD88 has also been identified as a target for miR-155 in the study of miR-155 expression [57]. In another study, miR-145 was found to target MAL, which is the bridging adaptor between TLR2- and TLR4-mediated MyD88-dependent signaling [58].

In addition, zinc finger proteins are among the most common regulatory factors in eukaryotes. A subclass of these proteins contains the recently identified BED finger DNA-binding domain, and these proteins are thought to function as either transcription activators or repressors by modifying the local chromatin structure through binding to GC-rich sequences [59, 60]. Mouse Zbed4, like its human ortholog, has 2 nuclear receptor-interacting motifs (LXXLL) characteristic of coactivators/corepressors of nuclear hormone receptors [60]. Zbed4 has been reported to interact with estrogen receptor alpha (ER*α*) and cellular myosin 9 (MYH9) in retinoblastoma cells [61]. However, association of Zbed4 with innate immunity has not yet been reported.

Although bioinformatics remains a helpful tool for predicting the targets of specific miRNAs, experimental validation by combined analysis of miRNA and mRNA expression provides conclusive evidence [62]. Investigation of the association between miRNA and mRNA expression on a genome-wide basis provides an analytical approach for identifying miRNA target genes [63–65]. However, such a combined approach might be still oversimplistic for relating miRNAs and their predicted targets primarily on the basis of the number of consensus sites in the 3′UTR because an exact match to the sequence of the seed region is not required. RIP-chip analysis combined with the Ago2 machinery is helpful for increasing specificity in calculating the correlation between miRNAs and mRNAs and identifying subsets of RNAs with related functions that are potentially coregulated in RNP complexes [66, 67]. While the RIP-chip assay can identify high-confidence miRNA targets, it may produce a smaller number of predicted targets after correlation with a stricter prediction algorithm, considering the accuracy and reproducibility of the whole genome array and the possibility that there might be some unidentified target genes repressed only during translation but not through mRNA degradation. Further gain-of-function or loss-of-function experiments will help in elucidating the exact roles of the identified target genes for each miRNA.

## 5. Conclusions

This study has profiled TLR4/NF-*κ*B-responsive miRNAs (miR-15a, miR-744, and miR-1196) in thigh skeletal muscle isolated following IRI and identified their potential target genes by using prediction algorithms and RNA-binding protein immunoprecipitation microarray profiling of Ago2 immunoprecipitated complexes. Although the exact roles of the IRI-upregulated miRNAs remain to be elucidated, this study provides novel insights into the epigenetic regulation in skeletal muscle following IRI.

## Supplementary Material

Supplementary File 1: The complete list of the significantly upregulated miRNA targets identified by a miRNA array in the muscles of C57BL/6 mice after ischemia and reperfusion.
Supplementary File 2: There was a general agreement of the expression of miRNA targets between microarray and qPCR results.

## Figures and Tables

**Table 1:** Significantly upregulated miRNA targets identified by a miRNA array in the muscles of C57BL/6 mice after 2 h of ischemia (Isc) and 0 h, 4 h, 1 d, or 7 d of reperfusion (Rep.) (detailed information in the Supplementary File 1).

Isc 2 h	Isc 2 h/Rep. 4 h	Isc 2 h/Rep. 1 d					Isc 2 h/Rep. 7 d		
log⁡_2_ ratio > 1	log⁡_2_ ratio > 1	log⁡_2_ ratio > 3	log⁡_2_ ratio > 2	log⁡_2_ ratio > 1			log⁡_2_ ratio > 2	log⁡_2_ ratio > 1	
miR-215	miR-493	miR-466d-5p	miR-467e	miR-1903	miR-542-5p	miR-370	miR-21	miR-214	miR-501-3p
		miR-466k	miR-466c-5p	miR-296-3p	miR-92a	miR-135a	miR-466d-3p	miR-466l	miR-199a-3p
		miR-466b-5p	miR-466j	miR-677	miR-292-5p	miR-218	miR-467b	miR-466f-3p	miR-464
		miR-468	miR-669d	miR-744	miR-764-3p	miR-770-3p	miR-551b	miR-669i	miR-698
		miR-466f-5p	miR-125b-3p	miR-669h-5p	miR-30b	miR-383	miR-467e	miR-466i	miR-582-3p
		miR-672	miR-16	miR-106b	miR-705	miR-674	miR-466g	miR-466a-3p	miR-488
		miR-669b	miR-669g	miR-683	miR-20b	miR-31	miR-297a	miR-31	miR-1186
		miR-669e	miR-696	miR-1196	miR-294	miR-190b	miR-467g	miR-669f	miR-330
		miR-466a-5p	miR-18b	miR-449b	miR-764-5p	miR-433	miR-467a	miR-574-3p	miR-335-3p
		miR-466h	miR-297a	miR-1194	miR-1907	miR-671-3p		miR-669h-3p	miR-222
		miR-210	miR-297c	miR-338-5p	miR-881	miR-682		miR-467f	miR-351
		miR-670	miR-493	miR-760	miR-1896	miR-450b-5p		miR-215	miR-346
		miR-467c	miR-467h	miR-1892	miR-423-5p	miR-743b-5p		miR-466b-3-3p	miR-467h
		miR-466e-5p	miR-297b-5p	miR-141	miR-691	miR-665		miR-142-3p	miR-878-3p
		miR-669a	miR-15a	miR-330	miR-7a	miR-667		miR-207	miR-717
			miR-669c	miR-689	miR-292-3p	miR-1898		miR-1192	miR-1196
			miR-467b	miR-547	miR-302c	miR-488		miR-713	miR-685
			miR-1188	miR-574-5p	miR-1893	miR-511		miR-674	miR-671-3p
			miR-122	miR-1187	miR-1224	miR-679		miR-199a-5p	miR-542-3p
			miR-711	miR-298	miR-302d	miR-453		miR-206	miR-669d
			miR-466f	miR-98	miR-214	miR-678		miR-706	miR-467b
			miR-214	miR-449c	miR-28	miR-615-5p		miR-1-2-as	miR-503
			miR-99b	miR-680	miR-710	miR-1897-5p		miR-1903	miR-1894-5p
			miR-465b-5p	miR-105	miR-291b-5p	miR-101a		miR-197	miR-877
			miR-346	miR-712	miR-685	miR-693-3p		miR-15b	miR-667
			miR-673-3p	miR-291a-5p	miR-877	miR-327			
			miR-681			miR-129-5p			
			miR-93						
			miR-1906						
			miR-1186						
			miR-546						
			miR-342-5p						
			miR-21						

**Table 2:** Tlr4/NF-*κ*B-responsive miRNA targets differentially expressed in the muscles of C57BL/6 and *Tlr*4^−/−^/*NF*-*κB* ^−/−^ mice after 2 h of ischemia and 1 d of reperfusion, identified by a miRNA array.

	Fold of expression (log⁡_2_)		
	C57BL/6	TLR4 KO mice	NF-*κ*B KO mice
miR-15a	2.63	−1.11	−1.10
miR-744	1.92	−2.36	−2.41
miR-1196	1.87	−1.78	−1.50

**Table 3:** Potential target genes for miR-15a, miR-744, and miR-1196 in the IRI muscles, identified by computational prediction combined with RNA-binding protein immunoprecipitation microarray profiling.

Accession	Gene	Definition
miR-15a		
NM_181412	Zbed4	Mus musculus zinc finger, BED domain containing 4
NM_199302	Lrsam1	Mus musculus leucine-rich repeat and sterile alpha motif containing 1
NM_153522	Scn3b	Mus musculus sodium channel, voltage-gated, type III, beta
NM_178378	Iqcg	Mus musculus IQ motif containing G
NM_008011	Fgfr4	Mus musculus fibroblast growth factor receptor 4

miR-744		
NM_181412	Zbed4	Mus musculus zinc finger, BED domain containing 4
NM_199302	Lrsam1	Mus musculus leucine-rich repeat and sterile alpha motif containing 1
NM_019553	Ddx21	Mus musculus DEAD (Asp-Glu-Ala-Asp) box polypeptide 21
NM_001024602	AW555464	Mus musculus expressed sequence AW555464

miR-1196		
NM_181412	Zbed4	Mus musculus zinc finger, BED domain containing 4
NM_177312	6330408A02Rik	Mus musculus RIKEN cDNA 6330408A02 gene
NM_134052	Adi1	Mus musculus acireductone dioxygenase 1
NM_001012450	Ankrd6	Mus musculus ankyrin repeat domain 6
NM_019553	Ddx21	Mus musculus DEAD (Asp-Glu-Ala-Asp) box polypeptide 21
NM_010164	Eya1	Mus musculus eyes absent 1 homolog (*Drosophila*)
NM_010517	Igfbp4	Mus musculus insulin-like growth factor binding protein 4
NM_001038609	Mapt	Mus musculus microtubule-associated protein tau
NM_008306	Ndst1	Mus musculus N-deacetylase/N-sulfotransferase (heparan glucosaminyl) 1
NM_001025613	Otud7b	Mus musculus OTU domain containing 7B
NM_145457	Paip1	Mus musculus polyadenylate binding protein-interacting protein 1
NM_183028	Pcmtd1	Mus musculus protein-L-isoaspartate (D-aspartate) O-methyltransferase domain containing 1
NM_018884	Pdzrn3	Mus musculus PDZ domain containing RING finger 3
NM_139269	Pla2g16	Mus musculus phospholipase A2, group XVI
NM_026164	Pnpla8	Mus musculus patatin-like phospholipase domain containing 8
NM_133485	Ppp1r14c	Mus musculus protein phosphatase 1, regulatory (inhibitor) subunit 14c
NM_001081347	Rhobtb1	Mus musculus Rho-related BTB domain containing 1
NM_177766	Slc35e1	Mus musculus solute carrier family 35, member E1
NM_175132	Synpo2l	Mus musculus synaptopodin 2-like
NM_027992	Tmem106b	Mus musculus transmembrane protein 106B
