# Supplementary material for: TLR4/NF-κB-Responsive MicroRNAs and Their Potential Target Genes: A Mouse Model of Skeletal Muscle Ischemia-Reperfusion Injury
Source: Biomed Res Int. 2015 Jan 26;2015:410721. doi: 10.1155/2015/410721 (PMC4321099; doi:10.1155/2015/410721)
Supplement: Supplementary file 1 — The complete list of the significantly upregulated miRNA targets identified by a miRNA array in the muscles of C57BL/6 mice after ischemia and reperfusion. Supplementary File 2: There was a general agreement of the expression of miRNA targets between microarray and qPCR results. [file 410721.f1.zip › Supplementary file 1.pdf]

ischemia 2 h			ischemia 2 h/reperfusion 4 h			ischemia 2 h/reperfusion 1 d			ischemia 2 h/reperfusion 7 d		
miRNA target	log2 (ratio)	P-value	miRNA target	log2 (ratio)	P-value	miRNA targets	log2 (ratio)	P-value	miRNA targets	log2 (ratio)	P-value
miR-215	1.02	2.40E-02	miR-493	1.07	4.11E-02	miR-466d-5p	3.53	2.22E-06	miR-21	2.37	1.37E-05
						miR-466k	3.37	3.61E-05	miR-466d-3p	2.21	2.43E-04
						miR-466b-5p	3.37	3.47E-06	miR-467b	2.14	7.79E-04
						miR-468	3.35	3.60E-06	miR-551b	2.14	6.38E-07
						miR-466f-5p	3.31	1.61E-06	miR-467e	2.07	3.02E-05
						miR-672	3.28	7.99E-07	miR-466g	2.05	3.16E-05
						miR-669b	3.24	1.31E-06	miR-297a	2.04	1.57E-04
						miR-669e	3.24	1.80E-07	miR-467g	2.04	1.69E-04
						miR-466a-5p	3.14	4.41E-07	miR-467a	2.03	3.93E-04
						miR-466h	3.14	2.09E-06	miR-214	1.95	6.32E-08

	miR-210	3.14	3.48E-07	miR-466l	1.94	2.93E-05
	miR-670	3.09	1.38E-07	miR-466f-3p	1.89	2.97E-04
	miR-467c	3.07	4.74E-08	miR-669i	1.89	4.12E-04
	miR-466e-5p	3.05	5.36E-06	miR-466i	1.87	3.54E-04
	miR-669a	3.04	9.93E-06	miR-466a-3p	1.84	4.67E-04
	miR-467e	2.96	1.09E-07	miR-31	1.83	7.77E-07
	miR-466c-5p	2.94	1.65E-07	miR-669f	1.79	7.29E-06
	miR-466j	2.89	2.39E-06	miR-574-3p	1.70	7.03E-05
	miR-669d	2.88	4.45E-06	miR-669h-3p	1.63	2.40E-04
	miR-125b-3p	2.88	1.22E-06	miR-467f	1.63	2.53E-04
	miR-16	2.87	3.89E-07	miR-215	1.62	1.46E-06
	miR-669g	2.87	3.29E-07	miR-466b-3-3p	1.59	6.23E-04
	miR-696	2.84	5.20E-08	miR-142-3p	1.57	4.98E-04

miR-18b	2.82	1.22E-06	miR-207	1.56	1.28E-02
miR-297a	2.81	1.21E-07	miR-1192	1.56	3.81E-04
miR-297c	2.76	3.16E-07	miR-713	1.55	6.39E-07
miR-493	2.72	1.02E-05	miR-674	1.54	4.15E-05
miR-467h	2.67	1.34E-03	miR-199a-5p	1.54	1.10E-04
miR-297b-5p	2.64	1.63E-04	miR-206	1.52	4.80E-05
miR-15a	2.63	7.94E-07	miR-706	1.51	7.21E-07
miR-669c	2.55	5.49E-06	miR-1-2-as	1.44	1.50E-03
miR-467b	2.50	1.66E-08	miR-1903	1.40	2.47E-03
miR-1188	2.45	5.90E-07	miR-197	1.34	1.55E-02
miR-122	2.44	4.77E-07	miR-15b	1.33	2.33E-06
miR-711	2.39	1.07E-13	miR-501-3p	1.33	8.54E-04
miR-466f	2.33	5.90E-04	miR-199a-3p	1.31	1.39E-04

miR-214	2.31	2.73E-09	miR-464	1.29	2.14E-06
miR-99b	2.26	4.80E-09	miR-698	1.25	9.13E-03
miR-465b-5p	2.24	9.09E-09	miR-582-3p	1.24	2.33E-07
miR-346	2.24	1.80E-06	miR-488	1.19	5.62E-05
miR-673-3p	2.17	3.84E-10	miR-1186	1.16	3.00E-05
miR-681	2.15	1.40E-05	miR-330	1.15	3.75E-04
miR-93	2.15	3.01E-07	miR-335-3p	1.14	9.88E-05
miR-1906	2.09	2.49E-07	miR-222	1.11	5.08E-04
miR-1186	2.05	1.55E-06	miR-351	1.10	8.81E-06
miR-546	2.02	1.60E-10	miR-346	1.10	9.96E-05
miR-342-5p	2.01	6.00E-07	miR-467h	1.08	5.51E-03
miR-21	2.00	5.37E-04	miR-878-3p	1.08	9.01E-04
miR-1903	1.98	1.16E-05	miR-717	1.08	1.48E-04

miR-296-3p	1.95	2.69E-06	miR-1196	1.06	5.18E-06
miR-677	1.94	4.57E-07	miR-685	1.05	1.91E-03
miR-744	1.92	1.02E-02	miR-671-3p	1.04	2.74E-05
miR-669h-5p	1.92	1.83E-06	miR-542-3p	1.03	2.17E-03
miR-106b	1.92	7.91E-07	miR-669d	1.02	6.27E-03
miR-683	1.90	7.77E-08	miR-467b	1.02	1.15E-03
miR-1196	1.87	5.61E-07	miR-503	1.02	5.28E-04
miR-449b	1.84	7.12E-08	miR-1894-5p	1.02	1.84E-04
miR-1194	1.81	3.67E-06	miR-877	1.02	4.16E-03
miR-338-5p	1.81	6.44E-07	miR-667	1.01	1.83E-04
miR-760	1.80	8.29E-12			
miR-1892	1.76	5.36E-18			
miR-141	1.73	1.24E-07			

miR-330	1.71	1.23E-08
miR-689	1.71	3.14E-03
miR-547	1.71	6.91E-06
miR-574-5p	1.68	1.80E-02
miR-1187	1.65	7.81E-03
miR-298	1.65	3.41E-11
miR-98	1.61	1.85E-06
miR-449c	1.59	2.43E-09
miR-680	1.55	1.16E-06
miR-105	1.53	3.82E-06
miR-712	1.53	4.17E-02
miR-291a-5p	1.52	1.07E-05
miR-542-5p	1.52	1.55E-03

miR-92a	1.52	5.54E-05
miR-292-5p	1.51	1.42E-03
miR-764-3p	1.51	2.77E-09
miR-30b	1.50	7.68E-06
miR-705	1.48	5.79E-07
miR-20b	1.48	1.41E-05
miR-294	1.47	1.54E-04
miR-764-5p	1.47	1.54E-05
miR-1907	1.45	9.34E-08
miR-881	1.44	3.04E-08
miR-1896	1.41	3.18E-09
miR-423-5p	1.41	1.22E-09
miR-691	1.40	8.02E-08

miR-7a	1.39	1.17E-06
miR-292-3p	1.37	4.71E-06
miR-302c	1.37	2.89E-05
miR-1893	1.33	1.28E-02
miR-1224	1.32	2.31E-03
miR-302d	1.31	2.92E-04
miR-214	1.31	3.79E-05
miR-28	1.27	3.83E-06
miR-710	1.27	1.93E-04
miR-291b-5p	1.27	7.62E-07
miR-685	1.26	4.75E-05
miR-877	1.26	9.67E-08
miR-370	1.25	2.81E-11

miR-135a	1.24	1.30E-05
miR-218	1.23	3.84E-05
miR-770-3p	1.22	4.55E-09
miR-383	1.21	3.55E-05
miR-674	1.21	3.13E-06
miR-31	1.20	7.27E-08
miR-190b	1.19	1.62E-04
miR-433	1.19	7.84E-07
miR-671-3p	1.16	4.30E-06
miR-682	1.16	2.21E-06
miR-450b-5p	1.14	1.07E-05
miR-743b-5p	1.14	2.16E-07
miR-665	1.14	6.95E-07

miR-667	1.13	7.14E-04
miR-1898	1.12	7.74E-04
miR-488	1.12	1.54E-05
miR-511	1.11	7.09E-06
miR-679	1.09	1.40E-04
miR-453	1.08	1.19E-04
miR-678	1.07	1.75E-06
miR-615-5p	1.07	7.36E-04
miR-1897-5p	1.04	6.48E-08
miR-101a	1.04	7.95E-08
miR-693-3p	1.02	6.49E-03
miR-327	1.02	2.44E-05
miR-129-5p	1.00	3.98E-06
