# Supplementary figures and images for: TLR4/NF-κB-Responsive MicroRNAs and Their Potential Target Genes: A Mouse Model of Skeletal Muscle Ischemia-Reperfusion Injury
Source: Biomed Res Int. 2015 Jan 26;2015:410721. doi: 10.1155/2015/410721 (PMC4321099; doi:10.1155/2015/410721)

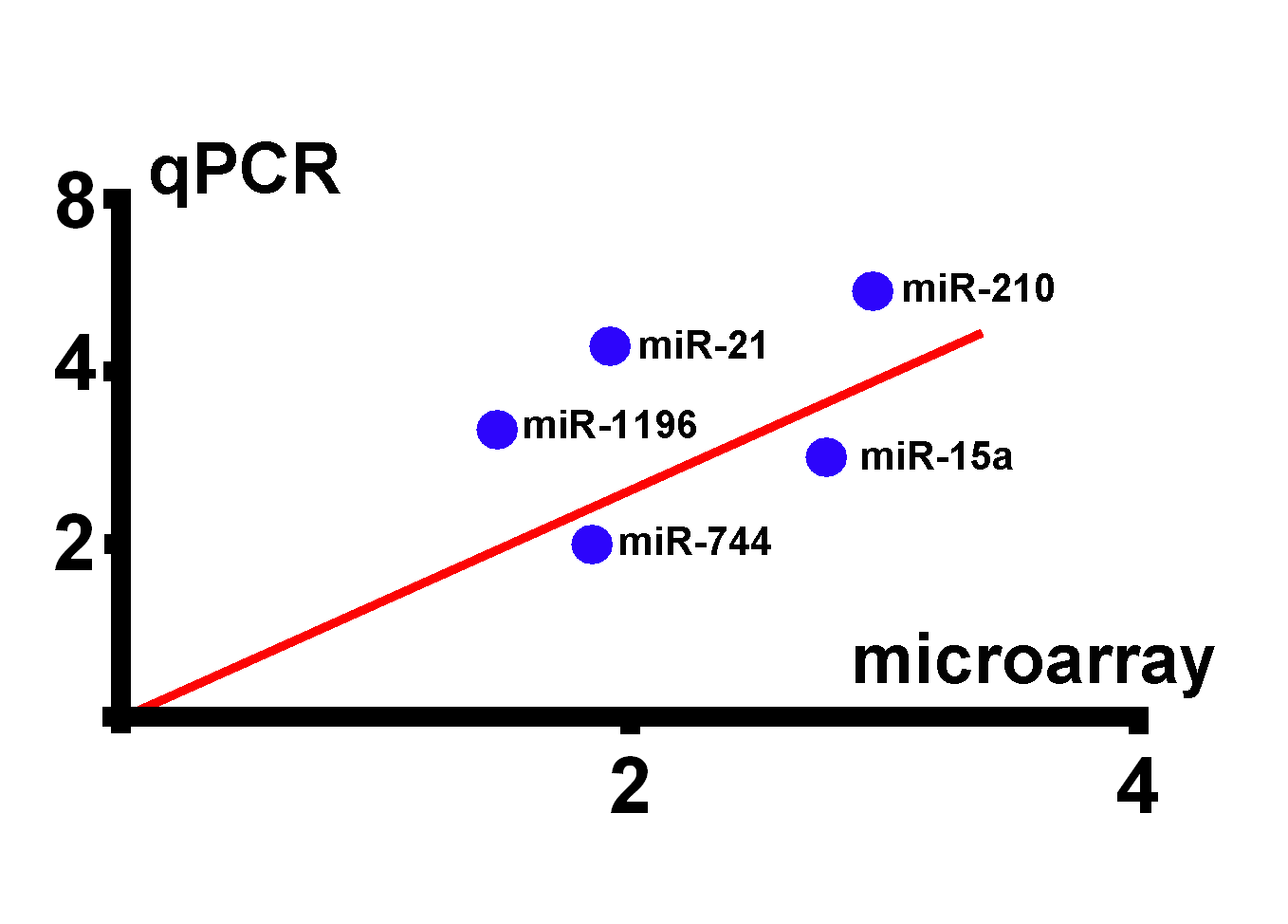

Supplement: Supplementary file 1 — The complete list of the significantly upregulated miRNA targets identified by a miRNA array in the muscles of C57BL/6 mice after ischemia and reperfusion. Supplementary File 2: There was a general agreement of the expression of miRNA targets between microarray and qPCR results. [file 410721.f1.zip › Supplementary file 2.tif]
